# Angiopoietin-Like 4 Regulates Epidermal Differentiation

**DOI:** 10.1371/journal.pone.0025377

**Published:** 2011-09-22

**Authors:** Mintu Pal, Ming Jie Tan, Royston-Luke Huang, Yan Yih Goh, Xiao Ling Wang, Mark Boon Yang Tang, Nguan Soon Tan

**Affiliations:** 1 School of Biological Sciences, College of Science, Nanyang Technological University, Singapore, Singapore; 2 National Skin Centre, Singapore, Singapore; University Hospital Hamburg-Eppendorf, Germany

## Abstract

The nuclear hormone receptor PPARβ/δ is integral to efficient wound re-epithelialization and implicated in epidermal maturation. However, the mechanism underlying the latter process of epidermal differentiation remains unclear. We showed that ligand-activated PPARβ/δ indirectly stimulated keratinocyte differentiation, requiring *de novo* gene transcription and protein translation. Using organotypic skin cultures constructed from PPARβ/δ- and angiopoietin-like 4 (ANGPTL4)-knockdown human keratinocytes, we showed that the expression of ANGPTL4, a PPARβ/δ target gene, is essential for the receptor mediated epidermal differentiation. The pro-differentiation effect of PPARβ/δ agonist GW501516 was also abolished when keratinocytes were co-treated with PPARβ/δ antagonist GSK0660 and similarly in organotypic skin culture incubated with blocking ANGPTL4 monoclonal antibody targeted against the C-terminal fibrinogen-like domain. Our focused real-time PCR gene expression analysis comparing the skin biopsies from wildtype and ANGPTL4-knockout mice confirmed a consistent down-regulation of numerous genes involved in epidermal differentiation and proliferation in the ANGPTL4-knockout skin. We further showed that the deficiency of ANGPTL4 in human keratinocytes and mice skin have diminished expression of various protein kinase C isotypes and phosphorylated transcriptional factor activator protein-1, which are well-established for their roles in keratinocyte differentiation. Chromatin immunoprecipitation confirmed that ANGPTL4 stimulated the activation and binding of JUNB and c-JUN to the promoter region of human involucrin and transglutaminase type 1 genes, respectively. Taken together, we showed that PPARβ/δ regulates epidermal maturation via ANGPTL4-mediated signalling pathway.

## Introduction

Adult epidermis is a stratified self-renewing epithelium in which keratinocytes in the basal and suprabasal layers cease to divide, concomitant with their outward movement, giving rise to differentiated cell layers of the spinous layer, granular layer and the stratum corneum. A tightly regulated homeostatic balance of epidermal cell proliferation and differentiation ensures proper epidermal structure and function [1], [2]. Transcriptional regulation plays an important role in skin maturation and abundant information is available on the various differentiation markers expressed in the epidermis [2], [3].

Nuclear hormone receptors, one of the largest known classes of transcription factors, have been implicated in skin development and maturation. Thyroid hormone, glucocorticoid, estrogen, vitamin D and retinoid X receptors, among others, were reported to either accelerate the maturation of the skin permeability barrier or modulate the differentiation of the epidermis [4], [5]. Of particular interest is the role of peroxisome proliferator–activated receptors (PPARs) β/δ isoform in epidermal differentiation and wound healing [6]–[10]. PPARβ/δ is an important regulator of keratinocyte survival in the wounded epidermis and is involved in cell adhesion and migration [11], [12]. A novel homeostatic control of keratinocyte proliferation was recently found, whereby PPARβ/δ regulates IL-1 signalling in dermal fibroblasts [13]. In addition to wound re-epithelialization, PPARβ/δ was also shown to stimulate epidermal differentiation [7], [10]. Our earlier results also confirmed a cell-autonomous action of PPARβ/δ in human keratinocyte differentiation [13]. However, the precise mechanism by which PPARβ/δ modulates epidermal differentiation remains obscure.

The adipocytokine angiopoietin-like 4 (ANGPTL4) represents a novel endocrine signal involved in the regulation of lipid and glucose metabolism, especially under fasting conditions [14]–[16]. The hypertriglyceridemic effect of ANGPTL4 is attributable to inhibition of lipoprotein lipase (LPL)-dependent very low density lipoprotein lipolysis by conversion of LPL dimers to monomers [17]. ANGPTL4 protects mice against the severe pro-inflammatory effects of dietary saturated fat in mesenteric lymph nodes by inhibiting macrophage LPL enzyme activity [18]. Podocyte-specific transgenic overexpression of hyposialylated ANGPTL4 induces proteinuria in glucocorticoid-sensitive nephrotic syndrome [19]. Recently, ANGPTL4 is shown to be important for cancer cell survival, where it sustains an elevated pro-survival intracellular O_2_^−^∶H_2_O_2_ ratio and confers anoikis resistance to tumor [20]. Effective cell-matrix communication is crucial for efficient wound healing. Recently, PPARβ/δ was shown to modify the wound microenvironment to coordinate cell-matrix communication by the upregulation of ANGPTL4. During wound healing, ANGPTL4 functions as a matricellular protein to coordinate cell-matrix communications by modulating integrin-mediated signaling pathway and intact matrix proteins availability which are essential for keratinocyte migration [21], [22]. Similar to PPARβ/δ, the expression of ANGPTL4 remains elevated after complete wound re-epithelialization. However, whether ANGPTL4 is involved in post-healing epidermal differentiation remains unknown. Herein, we showed that PPARβ/δ-mediated upregulation of ANGPTL4 expression in human keratinocytes stimulates the expression of protein kinase C (PKC) and activities of activator protein-1 (AP-1) transcription factors to modulate epidermal differentiation.

## Results

### PPARβ/δ modulates keratinocyte differentiation via an indirect mechanism

**Figure 1:**
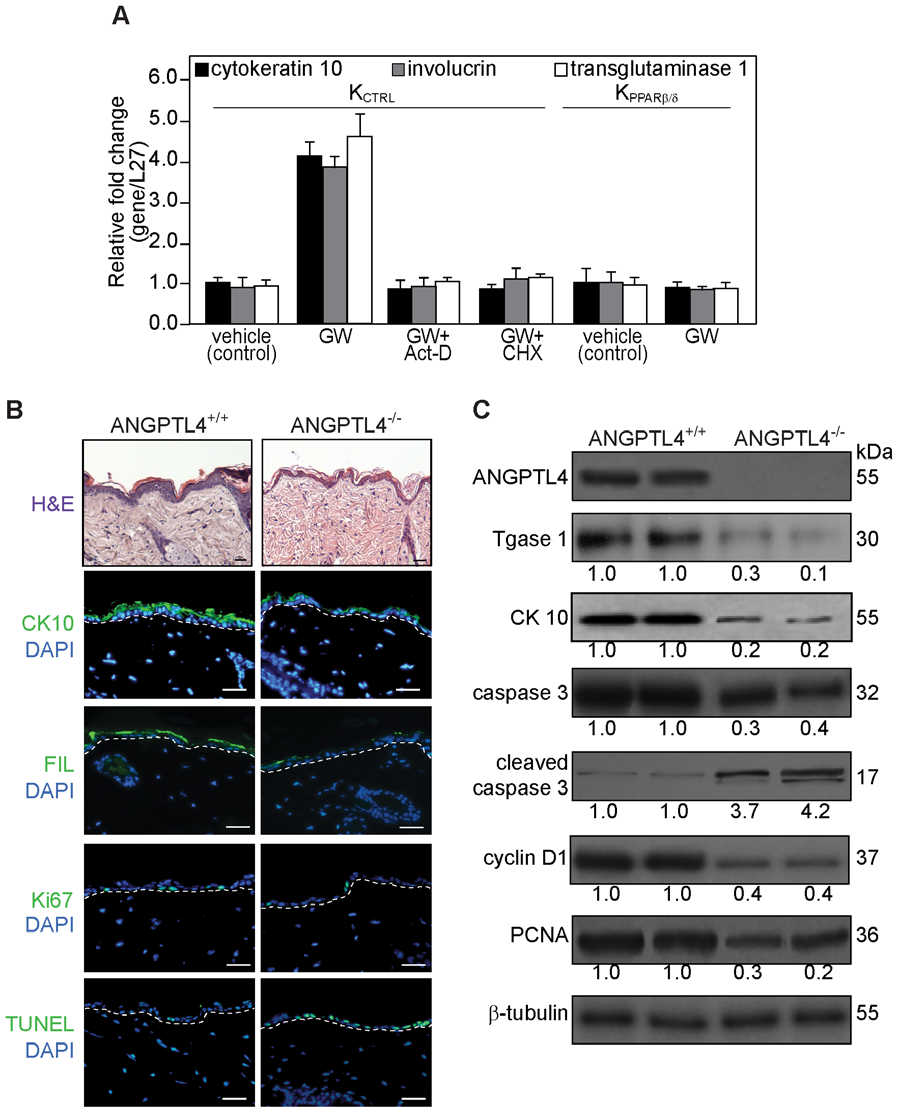
ANGPTL4 deficiency in mice impairs epidermal differentiation. (**A**) PPARβ/δ regulates keratinocyte differentiation requires *de novo* transcription and translation. Relative fold change in mRNA levels of cytokeratin 10, involucrin and transglutaminase type 1 in control (K_CTRL_) and PPARβ/δ-knockdown (K_PPARβ/δ_) human keratinocytes treated with either DMSO vehicle or PPARβ/δ agonist GW501516 (GW, 100 nM), in the absence or presence of RNA synthesis (actinomycin D, Act-D) or protein synthesis (cycloheximide, CHX) inhibitors as determined by quantitative real-time PCR. Act-D and CHX treatment alone did not affect the transcript level. Ribosomal protein L27 was used as a normalizing reference gene. Values are mean ± SEM of three independent experiments. (**B**) Haematoxylin and eosin (H&E) and immunofluorescence staining of skin biopsies from wildtype (ANGPTL4^+/+^) and ANGPTL4-knockout (ANGPTL4^−/−^) mice. Early (cytokeratin 10, CK10), late (fillaggrin, FIL) differentiation markers, proliferating (Ki67) and apoptotic (TUNEL) cells were identified using indicated antibodies or assay. White dotted lines indicated epidermis-dermis junctions. Sections were counterstained with DAPI (blue). Scale bars represent 40 µm. (**C**) Representative immunoblot of early differentiation (cytokeratin 10, CK10), terminal differentiation (transglutaminase type I, Tgase 1), proliferation (PCNA and cyclin D1), and apoptosis (cleaved caspase 3) markers in ANGPTL4+/+ and ANGPTL4−/− skin biopsies. Immunoblot data are from three independent experiments performed in duplicate. β-tubulin serves as a loading and transfer control.

Ligand-activated PPARβ/δ stimulates keratinocyte differentiation by a cell-autonomous mechanism [13]. In the first instance, we determine if PPARβ/δ directly regulates keratinocyte differentiation. Human primary keratinocytes were treated with 100 nM of GW501516 (GW) in the presence or absence of cycloheximide or actinomycin D. GW501516 is a selective PPAR β/δ agonist [23]. The mRNA levels of differentiation markers cytokeratin 10, involucrin and transglutaminase 1 were increased in GW-treated keratinocytes, consistent with previous observations [10]. The increased mRNA levels induced by GW were abolished in actinomycin- and cycloheximide-treated cells, suggesting that ligand-activated PPARβ/δ required *de novo* gene transcription and protein translation to stimulate keratinocytes differentiation. The pro-differentiating effect of GW was absent in PPARβ/δ-deficient (K_PPARβ/δ_) keratinocytes indicating that GW mediates its effect via PPARβ/δ (Figure 1A). K_PPARβ/δ_ cells were obtained as previously described [13]. As expected, the mRNA level of ANGPTL4, a PPARβ/δ target gene, was increased by GW treatment, and abolished in actinomycin- but not cycloheximide-treated cells, as previously observed [14], [24].

### ANGPTL4 deficiency results in impaired epidermal differentiation

**Figure 2:**
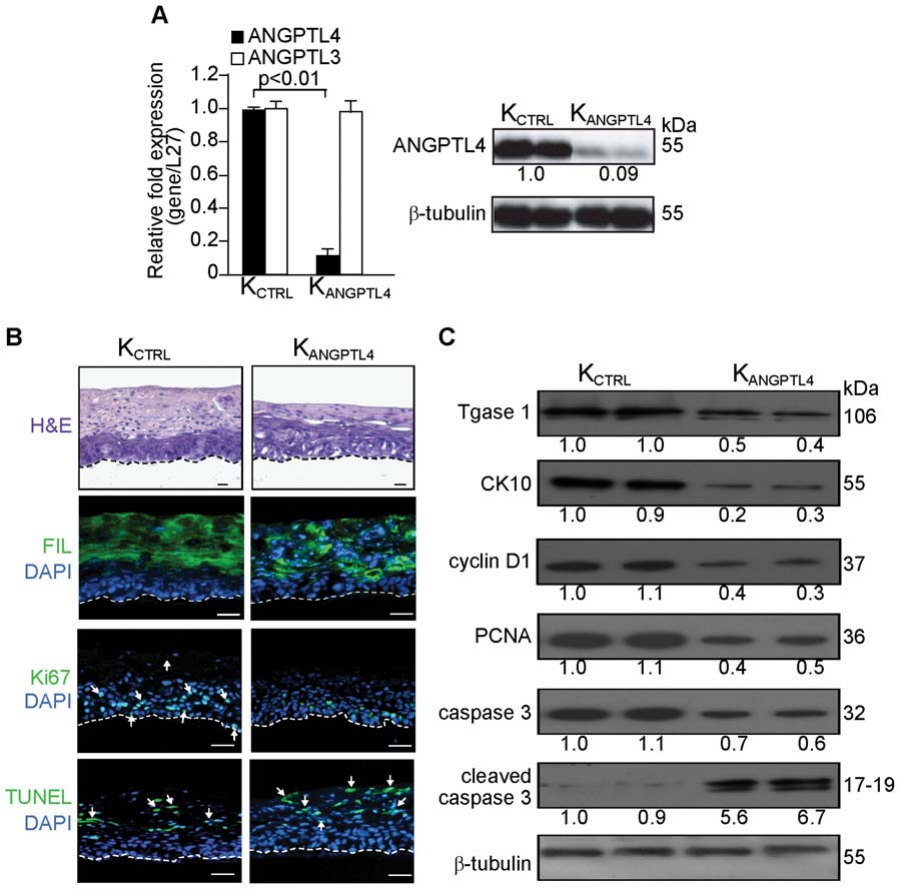
Organotypic skin coculture (OTC) of ANGPTL4-deficient human primary keratinocytes displayed impaired epidermal differentiation. (**A**) Relative mRNA and/or protein levels of ANGPTL4 and ANGPTL3 in human primary keratinocytes transduced with either scrambled control (K_CTRL_) or ANGPTL4 siRNA (K_ANGPTL4_). Values below immunoblot bands represent the mean fold differences in protein expression level when compared with K_CTRL_ from 3 independent experiments. (**B**) Haematoxylin and eosin (H&E) and immunofluorescence staining of OTC sections constructed with either control (K_CTRL_) or ANGPTL4-knockdown (K_ANGPTL4_) human keratinocytes. Late (fillaggrin, FIL) differentiation markers, proliferating (Ki67) and apoptotic (TUNEL) cells were identified using indicated antibodies or assay. White dotted lines indicated epidermis-dermis junctions. Sections were counterstained with DAPI (blue). Scale bars represent 40 µm. (**C**) Representative immunoblot of early epidermal differentiation (cytokeratin 10, CK10), terminal differentiation (transglutaminase type I, Tgase 1), proliferation (PCNA and cyclin D1), and apoptosis (cleaved caspase 3) markers in isolated epidermis of indicated OTCs. All immunoblot data are from three independent experiments performed in duplicate. β-tubulin serves as a loading and transfer control.

To examine if ANGPTL4 plays a role in epidermal differentiation, we first examine the skin biopsies from ANGPTL4-null (ANGPTL4^−/−^) and wildtype (ANGPTL4^+/+^) mice [25]. The deficiency in ANGPTL4 resulted in thinner epidermis (ANGPTL4^+/+^ vs ANGPTL4^−/−^: 32.5±12.4 vs 21.9±4.6 µm, p<0.01, n = 8) (Figure 2A). Immunofluorescence (IF) staining using differentiation markers, cytokeratin 10 and filaggrin showed that ANGPTL4^−/−^ had an impaired epidermal differentiation when compared with ANGPTL4^+/+^ (Figure 1B). Immunoblot analysis using differentiation markers, cytokeratin 10 and transglutaminase 1, further confirmed our findings (Figure 1B). ANGPTL4^−/−^ epidermis also showed more apoptotic (TUNEL-positive) (ANGPTL4^+/+^ vs ANGPTL4^−/−^: 4±1.7 vs 12±3.4 labeled cells per microscopic field; p<0.01, n = 8) and reduced Ki67-positive proliferating cells as compared to the control wildtype (ANGPTL4^+/+^ vs ANGPTL4^−/−^: 15±1.1 vs 4±0.7; p<0.01, n = 8) (Figure 1B). These were further supported by immunoblotting with cyclin D1 and PCNA as proliferation markers, as well as with cleaved caspase 3 as an apoptotic marker (Figure 1C). Lending additional support, our focused real-time PCR gene expression analysis comparing the skin biopsies from ANGPTL4^+/+^ and ANGPTL4^−/−^ mice confirmed a consistent down-regulation of numerous genes involved in the differentiation and proliferation of ANGPTL4^−/−^ skin (Table S1).

To further strengthen our findings, we performed similar analysis using organotypic skin culture (OTC) which closely mimics epidermal regeneration [13], [26]. We first suppressed endogenous ANGPTL4 expression using a lentivirus-mediated ANGPTL4 siRNA in the primary human keratinocytes as previously described [21], [22]. The ANGPTL4 expression level in ANGPTL4-knockdown keratinocytes (K_ANGPTL4_) was reduced by 90% compared with scrambled control-siRNA keratinocytes (K_CTRL_). No detectable change in the mRNA level of angiopoietin-like 3 (ANGPTL3), a member of the angiopoietin-like protein family that has the highest sequence similarity to ANGPTL4, indicating the specificity of the knockdown (Figure 2A). The specificity of anti-ANGPTL4 antibody was previously verified [21].

In OTC, either K_CTRL_ or K_ANGPTL4_ keratinocytes were seeded on a dermal fibroblast-embedded collagen matrix and cultured at air-exposed interface to induce stratification and differentiation. Consistent with the above findings from the mice skin biopsies, haematoxylin and eosin stain revealed that the epidermis was thinner in K_ANGPTL4_ than K_CTRL_ (K_ANGPTL4_ vs K_CTRL_: 248.7±25.1 vs 328.9±27.4 µm, p<0.01, n = 6) (Figure 2B). IF staining and immunoblot analysis using differentiation markers cytokeratin 10, filaggrin and transglutaminase 1 showed that K_ANGPTL4_ OTCs had an impaired epidermal differentiation when compared with K_CTRL_ (Figure 2B & C). K_ANGPTL4_ OTCs also showed more apoptotic (TUNEL-positive) (K_ANGPTL4_ vs K_CTRL_: 53±2.7 vs 18±2.5 labeled cells per microscopic field; p<0.01, n = 6) and reduced Ki67-positive proliferating cells as compared to K_CTRL_ OTCs (K_ANGPTL4_ vs K_CTRL_: 18±2.9 vs 31±8.1; p<0.01, n = 6) (Figure 2B). These were further supported by immunoblotting with cyclin D1 and PCNA as proliferation markers, as well as with cleaved caspase 3 as an apoptotic marker (Figure 2C). Altogether, these results suggest that ANGPTL4 modulates epidermal differentiation.

### ANGPTL4 protein induces keratinocyte differentiation

**Figure 3:**
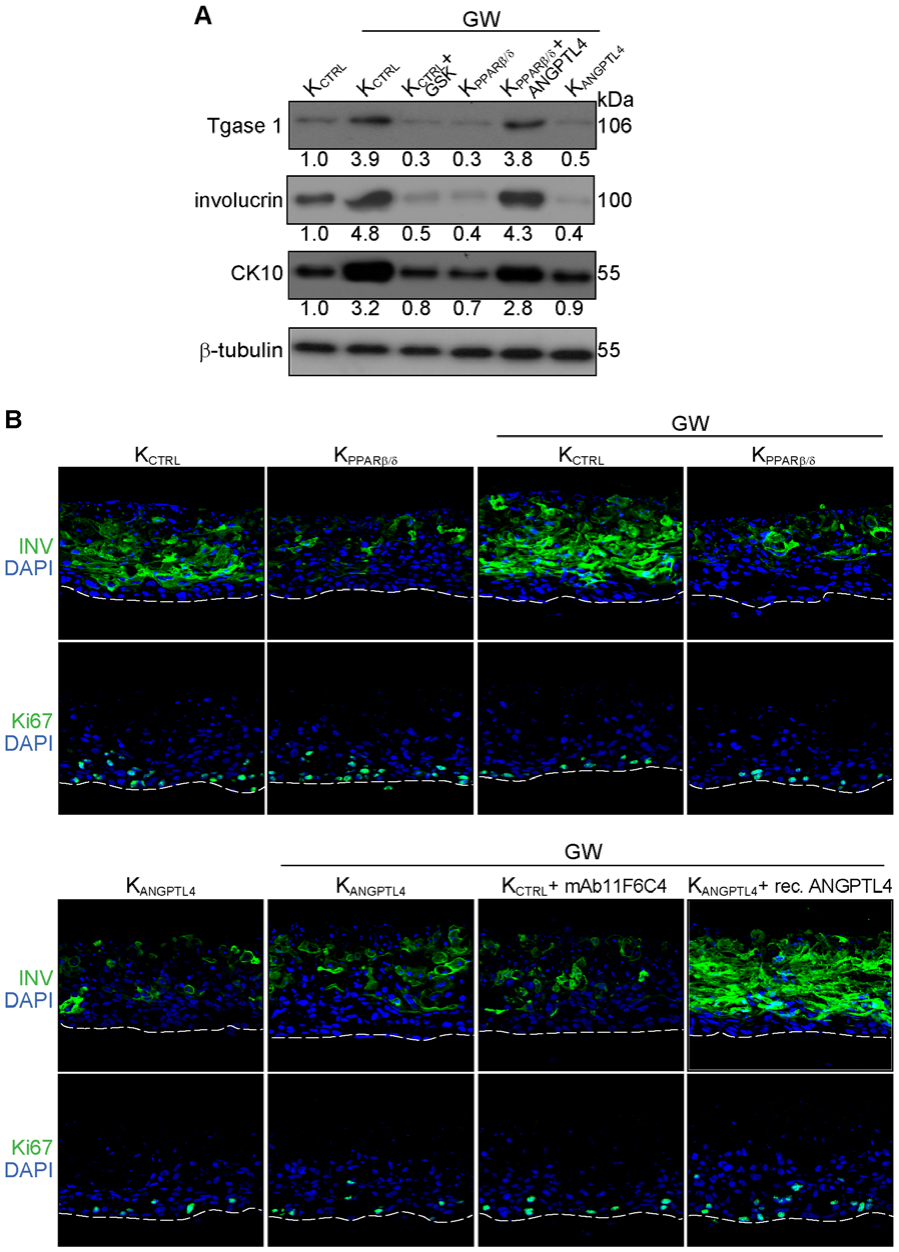
PPARβ/δ modulates epidermal differentiation via ANGPTL4. (**A**) Immunoblot analysis of early (cytokeratin 10, CK 10), late (involucrin) and terminal (transglutaminase type I, Tgase 1) epidermal differentiation markers in K_CTRL_, K_PPARβ/δ_ or K_ANGPTL4_ keratinocytes. (**B**) Immunofluorescence staining of late epidermal differentiation (involucrin, INV) and proliferation (Ki67) markers of either K_CTRL_-, K_PPARβ/δ_- or K_ANGPTL4_-dervied OTC sections, subjected to indicated treatments. PPARβ/δ agonist GW501516 (GW, 100 nM), PPARβ/δ antagonist GSK0660 (GSK, 0.5 µm), recombinant ANGPTL4 (rec. ANGPTL4, 6 µg/ml) and blocking ANGPTL4 monoclonal antibody (mAb11F6C4, 6 µg/ml). mAb11F6C4 targets the C-terminal fibrinogen-like domain of ANGPTL4 and has been shown to block ANGPTL4 interaction with integrin β1/β5. Immunoblot data are from three independent experiments performed in duplicate. β-tubulin serves as a loading and transfer control. White dotted lines indicated epidermis-dermis junctions. Sections were counterstained with DAPI (blue). Scale bars represent 40 µm.

ANGPTL4 is a direct transcriptional target gene of PPARβ/δ in murine and human keratinocytes. As a novel matricellular protein, ANGPTL4 may play an important role in cellular proliferation and differentiation [21], [22]. To investigate if ANGPTL4 is required for PPARβ/δ-mediated keratinocyte differentiation, we first examine the expression of differentiation markers on GW-treated K_CTRL_, K_PPARβ/δ_ and K_ANGPTL4_ keratinocytes. Consistent with our above findings, GW-activated PPARβ/δ induced keratinocyte differentiation as evidenced by the increased expression of transglutaminase type I, involucrin and cytokeratin 10 (Figure 3A), which was diminished either upon co-treatment with selective PPARβ/δ antagonist GSK0660 [27] or in K_PPARβ/δ_, as well as in K_ANGPTL4_ (Figure 3A). Notably, the differentiation potential of K_PPARβ/δ_ was restored by exogenous recombinant ANGPTL4 protein (Figure 3A). To further strengthen our finding, we subjected OTCs to various indicated treatments and examined epidermal differentiation by immunostaining. Comparing K_PPARβ/δ_ and K_CTRL_-derived OTCs, our results confirmed that GW mediated its pro-differentiation effect via PPARβ/δ (Figure 3B, upper panel). ANGPTL4-deficient keratinocytes also exhibited impaired epidermal differentiation regardless of GW treatment (Figure 3B, lower panel). Epidermal differentiation was also attenuated upon co-treatment with neutralizing monoclonal anti-ANGPTL4 antibodies (mAb11F6C4) (Figure 3B, lower panel), which was shown previously to block the interaction of ANGPTL4 with either specific integrins or extracellular matrix proteins [20]–[22]. Consistent with the pro-differentiation role of ANGPTL4, exogenous recombinant ANGPTL4 stimulated epidermal differentiation in K_ANGPTL4_-derived OTCs. These observations suggested a pivotal role of ANGPTL4 in PPARβ/δ-mediated keratinocyte differentiation.

### ANGPTL4 modulates PKC and AP-1 dependent signaling pathways

**Figure 4:**
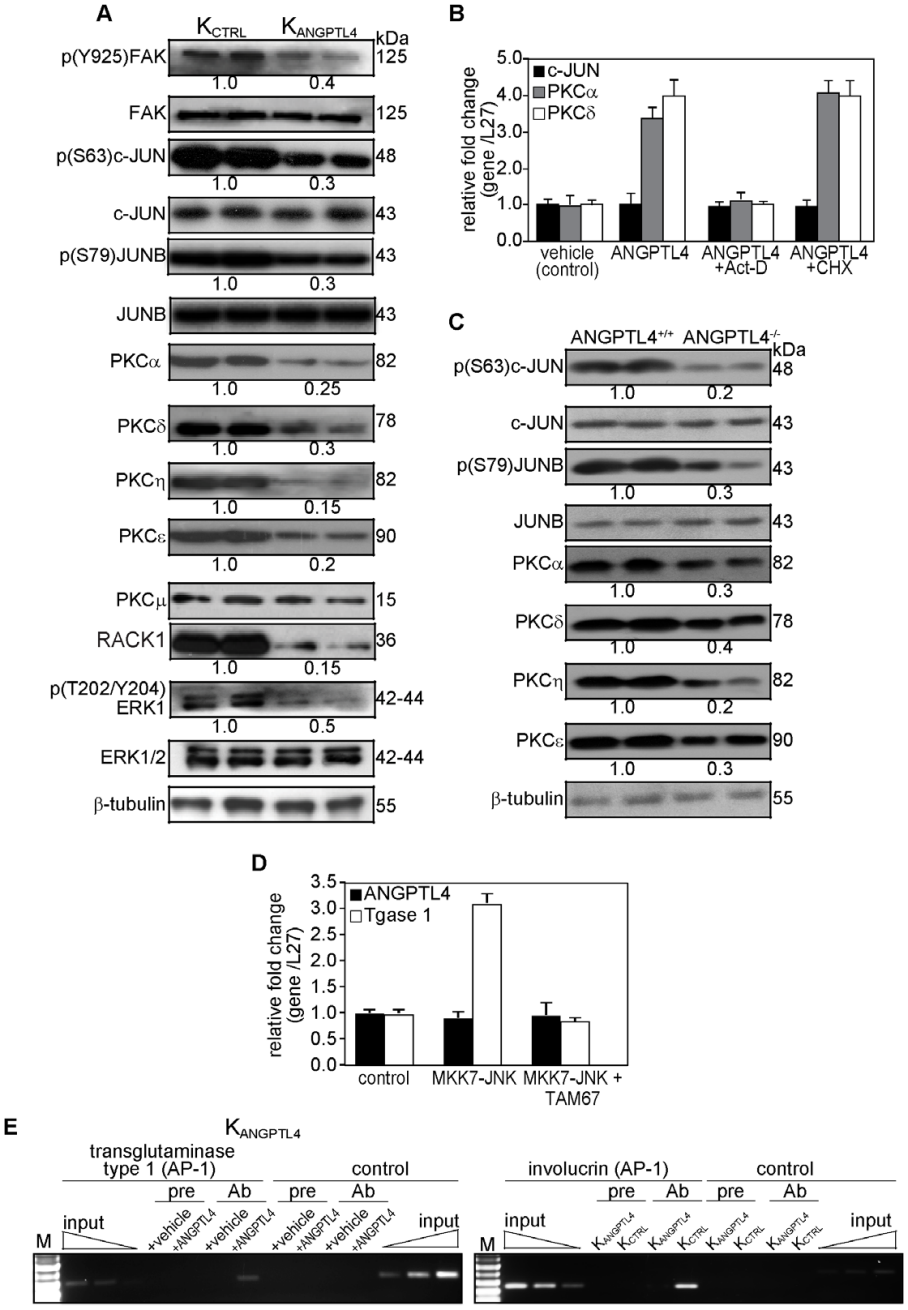
ANGPTL4 modulates the expression of PKCs and activities of AP-1. (**A and C**) Representative immunoblot analysis of indicated proteins from (**A**) epidermis of K_CTRL_- and K_ANGPTL4_-derived OTCs or (**C**) skin biopsies of ANGPTL4^+/+^ and ANGPPTL4^−/−^ mice. Values below immunoblot bands represent the mean fold differences in protein expression levels when compared with either K_CTRL_ (for A) or ANGPTL4^+/+^ (for C), which was assigned the value one, from 3 independent experiments. β-tubulin serves as a loading and transfer control. (**B**) Relative fold change in mRNA levels of c-JUN, PKCα and PKCδ in control (K_CTRL_) human keratinocytes treated with either DMSO vehicle or recombinant ANGPTL4 (6 µg/ml), in the absence or presence of RNA synthesis (actinomycin D, Act-D) or protein synthesis (cycloheximide, CHX) inhibitors as determined by quantitative real-time PCR. Act-D and CHX treatment alone did not affect the transcript level. (**D**) Relative fold change in mRNA levels of ANGPTL4 and transglutaminase type I in human keratinocytes transiently transfected with expression vectors encoding for either MMK7-JNK or TAM67. Empty expression vector was used a scontrol. Ribosomal protein L27 was used as a normalizing reference gene. Values are mean ± SEM of three independent experiments. (**E**) Chromatin immunoprecipitations were done either with vehicle (PBS)- and recombinant ANGPTL4-treated K_ANGPTL4_ or with K_CTRL_ and K_ANGPTL4_ keratinocytes using pre-immune IgG (pre), antibody against phospho-cJUN and phospho-JUNB (Ab). The AP-1 binding site in the human transglutaminase type 1 and involucrin promoter region were immunoprecipitated and specifically amplified using phospho-cJUN and phospho-JUNB, respectively. No amplified signal was obtained in vehicle-treated K_ANGPTL4_, K_ANGPTL4_ or using pre-immune IgG. A control region upstream of the AP-1 binding site served as negative control. Aliquots of the chromatin were also analyzed before immunoprecipitation (input). M: 100-bp DNA marker.

The activation of PKC and various members of transcriptional factor AP-1 are important for the expression of different keratinocyte differentiation markers [28], [29]. Differentiation-promoting agents have been shown to regulate the expression of differentiation marker genes via activation of PKC-dependent signaling pathway that targets AP-1 proteins. ANGPTL4 interacts with specific integrins and their cognate ligands to activate integrin-mediated signaling [20]–[22]. To gain more insight into ANGPTL4-mediated signaling pathways for keratinocyte differentiation, we performed immunoblot analysis of indicated intracellular signaling mediators. Consistent with the notion of integrin activation, the expression of phosphorylated FAK was reduced in K_ANGPTL4_ as compared with K_CTRL_ (Figure 4A). Our immunoblot also showed an attenuated expression of classical and novel PKC isoforms, namely PKCα, δ, ε and η in K_ANGPTL4_, compared with K_CTRL_ (Figure 4A). We also detected a diminished level of phosphorylated ERK-1/2 in K_ANGPTL4_, which has been shown to down-regulate PKCδ [30]. K_ANGPTL4_ also exhibited decreased expression of RACK1 [31], indicating attenuated PKC-mediated signal transduction (Figure 4A). The expression of PKCμ appeared slightly reduced in K_ANGPTL4_ when compared with with K_CTRL_, albeit not statistically significant (Figure 4A). The dysregulation of PKCs would have an influence on the activation of AP-1 proteins and subsequently keratinocyte differentiation [26], [28]. Indeed, our immunoblot analysis showed reduced phosphorylated i.e. activated c-JUN and JUNB (Figure 4A). To examine if ANGPTL4 has a direct effect on the expression of these signaling proteins, we examined their mRNA levels in K_ANGPTL4_ treated with recombinant ANGPTL4 in the presence of either actinomycin D or cycloheximide. The increased mRNA levels of PKCα and PKCδ induced by ANGPTL4 were abolished in actinomycin D- but not cycloheximide-treated cells, suggesting a transcriptional regulatory mechanism. Interestingly, no difference in c-JUN mRNA level was detected in all tested conditions, indicating a post-translation mechanism, most likely phosphorylation (Figure 4B). Similarly changes in total or phosphorylated protein expression level was also observed in the skin biopsies of ANGPTL4^+/+^ and ANGPTL4^−/−^ mice (Figure 4C).

The human ANGPTL4 gene is transcriptionally regulated by PPARβ/δ and HIFα. Indeed, functional PPAR response elements and HIF binding site have been identified [24], [32]. It was also reported that phorbol ester regulates ANGPTL4 expression in human smooth muscle cells [33]. In addition, several putative AP-1 binding sites were observed on the human ANGPTL4 promoter [33]. Thus, we question if AP-1 can regulates ANGPTL4 gene transcription in keratinocytes. To this end, we examine the expression level of ANGPTL4 mRNA in keratinocytes transfected with expression vector encoding for either MKK&-JNK or TAM67, a dominant negative AP-1. The expression of MKK7-JNK fusion proteins led to constitutive activation of JNK through intramolecular phosphorylation by MKK7, and increases AP-1 activity [28], [34]. As positive control, the level of transglutaminase type 1 mRNA was used. As expected, keratinocytes transfected with expression vector for MKK7-JNK showed a 3.5-fold increase in the mRNA level of transglutaminase type 1, which was abolished when cells were co-transfected with TAM67 (Figure 4D). No significant change in ANGPTL4 mRNA was observed in keratinocytes under all examined conditions, indicating that its expression was not regulated by AP-1 at least in keratinocyte (Figure 4D).

### ANGPTL4 increases activated AP-1 binding on the promoters of human involucrin and transglutaminase type 1 gene

Finally, to determine whether ANGPTL4-stimulated AP-1 activation was associated with changes in the transcription regulation of AP-1 dependent differentiation protein markers, we performed chromatin immunoprecipitation (ChIP) using either phospho-c-JUN or JUNB. Our ChIP showed that phospho-JUNB specifically bound to the AP-1 responsive elements in the promoter of human involucrin gene in K_CTRL_ but not in K_ANGPTL4_. Similarly, ChIP using phospho-c-JUN antibody showed that phosphorylated c-JUN was bound to the AP-1 responsive elements in promoter of human transglutaminase type 1 gene in K_ANGPTL4_ treated with recombinant ANGPTL4, when compared with vehicle (PBS) (Figure 4E). No immunoprecipitation and amplification were seen with pre-immune IgG and with a control sequence upstream of the AP-1 element in the promoters of transglutaminase type 1 and involucrin gene (Figure 4E). Altogether, these results indicate that GW-activated PPARβ/δ mediates keratinocyte differentiation, at least through a mechanism that involves the transcriptional regulation of ANGPTL4 and subsequent activation of PKCs and AP-1 transcription factors.

## Discussion

Epidermal maturation involves keratinocyte differentiation which is crucial to protect the organism from dehydration and to defense against microbial, mechanical, chemical and UV aggression. Here we provide a mechanism for the role of ANGPTL4, PPARβ/δ target gene, in epidermal differentiation via AP-1-dependent signaling pathways. We noted that the pro-differentiating effect of ligand-activated PPARβ/δ is cell-autonomous and mediated by an indirect mechanism. This is consistent with earlier studies using organotypic skin coculture [13] and that the increase in the expression of differentiation-related proteins was a late events, appearing only after 48 h of treatment [10]. Careful analysis of organotypic skin cocultures constructed using PPARβ/δ and ANGPTL4-deficient human keratinocytes, immunoblotting, as well as reconstitution and immuno-depletion experiments revealed that the autonomous regulation of epidermal differentiation by PPARβ/δ is mediated via ANGPTL4 and its intracellular modulation through an integrin-mediated signaling. Our observation suggested that ANGPTL4 stimulated the increase activation of c-JUN and JUNB, while it transcriptionally regulated the expression of PKCs. Indeed, chromatin immunoprecipitation further confirmed that differentiation markers like transglutaminase 1 and involucrin were induced by ligand activated-PPARβ/δ and ANGPTL4, associated with increased AP-1 binding to the cognate promoter. Immunoblotting analysis also showed a decrease in the expression of classical and novel PKC isoforms in ANGPTL4-deficient epidermis. The role of PKCs in the epidermal differentiation is well-established [28]. Both the PPARβ/δ- and ANGPTL4- knockout mice did not display any obvious skin abnormalities at normal situation. However, these mutant mice exhibited impaired wound repair and displayed altered epidermal differentiation, indicating that their role is context-dependent such as wound healing. This is also consistent with the role of ANGPTL4 as a matricellular protein, which has a distinguishing characteristic that it is expressed at high levels in response to injury [35].

Early studies have provided strong evidence for the involvement of PPARβ/δ in the different phases of wound healing process [8], [9]. The expression of PPARβ/δ is upregulated in adult epidermis by inflammatory stimuli during skin injury, which also provokes keratinocyte activation [6], [7]. Further studies showed that the activation of PPARβ/δ conferred an anti-apoptotic effect on the keratinocytes *in vivo*, hence protecting them from cytokine-induced apoptosis during the inflammatory phase of wound repair, thereby maintaining a sufficient number of viable migratory keratinocytes. PPARβ/δ activity also amplifies the response of keratinocytes to a chemotactic signal, promotes integrin recycling in wound keratinocytes and stimulates the production of ANGPTL4 in wound epithelia to modulate the wound microenvironment and thereby favors cell migration for re-epithelialization phase of the wound healing process [7], [11], [22]. Our result herein provides new insights into the mechanism by which PPARβ/δ-mediated ANGPTL4 to regulate PKCs and AP-1 transcription factor for epidermal differentiation, suggest that it may also be important for the maturation of the epidermis consequently its functional integrity in the remodeling phase of wound healing. Our study also provides a novel role of ANGPTL4 that will be of value to future investigation of transcriptional networks involved in complex epithelia development and differentiation.

The role of ANGPTL4 in the differentiation of other cell types remains unclear, although several evidences suggested that ANGPTL4 may be involved in or associated with adipose differentiation, endothelial cell growth and tubule formation. Hormone-dependent adipocyte differentiation coincided with a dramatic early induction of the ANGPTL4 transcript. The ANGPTL4 gene was expressed in mouse 3T3-L1 adipocytes before and after differentiation, the level increasing post-differentiation [16], [24], [36]. However, the in vivo role of ANGPTL4 in adipocyte maturation is complicated as transgenic ANGPTL4 or knockout mice showed that the maintenance of normal fat mass was a result from compensatory metabolic changes in adipose triglyceride metabolism [25]. It was reported that phornol ester and PDGFα induced the mRNA and 4 in several cell types of the lung. They revealed that this induction was mediated via PKC, ERK and JNK pathways. Importantly, they proposed that this induction of ANGPTL4 through the activation of PKC may play an important role in the regulation of airway remodeling and lipid homeostasis [33]. The role of ANGPTL4 on tubule formation of endothelial cells remains controversial. *In vitro* experiments using purified recombinant ANGPTL4 protein revealed that ANGPTL4 markedly inhibited the proliferation, chemotaxis and tubule formation of endothelial cells [37], [38]. However, other reports found that ANGPTL4 stimulated endothelial cell growth and tubule formation particularly in neovascularization of adipose tissue to support increased adipocyte number [39]. In support, we also showed reduced angiogenesis in ANGPTL4^−/−^ mice during skin wound repair when compared with ANGPTL4^+/+^ mice [21], [22]. Clearly, these observations justify further investigations on the role of ANGPTL4 in differentiation of other cell types.

## Materials and Methods

### Reagents and antibodies

Antibodies used: Ki67, cytokeratin 10 (CK10) and filaggrin (NovoCastra); Alexa488- or Alexa594-conjugated secondary antibodies (Molecular Probes); β-tubulin, cyclin D1, PCNA, RACK-1, transglutaminase 1 (Tgase 1), ERK-1, p(T202/Y204)-ERK1/ERK2 and HRP-conjugated secondary antibodies (Santa Cruz Biotechnology); PKC isotypes (BD Biosciences); FAK, caspase 3 and cleaved caspase 3 (Cell Signaling); polyclonal antibodies against the C-terminal fibrinogen-like region mouse of ANGPTL4 were produced in-house. Rat tail collagen type I was obtained from BD Biosciences. Primary neonatal human fibroblasts and keratinocytes were obtained from Invitrogen. Otherwise stated all chemicals were from Sigma-Aldrich.

### Organotypic skin culture (OTC)

Primary human keratinocytes and fibroblasts were routinely maintained in defined keratinocyte growth medium (EpiLife; Invitrogen) and medium 106, respectively, as described by the manufacturer. OTCs were performed as previously described [13].

### Immunofluorescence

Tissues or OTCs were fixed with 4% paraformaldehyde in PBS for 2 h at 25°C. The fixed OTCs were washed twice with PBS and embedded in Tissue-Tek OCT freezing compound medium (Sakura). 10 µm cryostat tissue sections were mounted on SuperFrost Plus slides (Menzel-Glaser). The sections were processed for immunofluorescence as previously described [40]. The apoptotic keratinocytes were detected using the TUNEL assay according to the manufacturer's protocol (Roche). As positive control for the TUNEL assay, the section was pretreated with DNase I. The slides were mounted with antifade reagent (ProLong Gold; Invitrogen) with DAPI. Images were taken using a Zeiss LSM 710 confocal microscope with a 40× objective and ZEN 2009 software.

#### Animal Experiment

Pure-bred wild type (ANGPTL4+/+) and ANGPTL4-knockout (ANGPTL4−/−) mice on a C57Bl/6 background were used [25]. All mice used in this study were individually caged, house in a temperature-controlled room (23°C) on a 10-h dark/14-h light cycle, and fed with the standard mouse chow diet. A full thickness mid-dorsal skin biopsy (0.5-cm^2^) was excised from 8-week old male mice. The biopsy was either processed for immunofluorescence staining or snap frozen in liquid nitrogen for protein extraction and subsequent immunoblot analysis. Animal experiments were approved by the University Institutional Animal Care and Use Committee (ARF-SBS/NIE-A-0093, -0078, and -004) and Biological Safety Committee (BPN-0004-2011-SBS). Hematoxylin and eosin (H&E) stained images and histomorphometric measurements were taken using using MIRAX MIDI with Plan-Apochormatic 20×/0.8 objectives using MIRAX Scan software (Carl Zeiss). Epidermal thickness was obtained from three independent skin biopsies using TissueQuest software (TissueGnostics GmbH) [21].

### Knockdown of ANGPTL4

siRNA against human ANGPTL4 and scrambled sequence as control were subcloned into the pFIV-H1/U6-puro siRNA lentivirus system. The correct pFIV siRNA construct was verified by sequencing using H1 primer. The sequence of the siRNAs was as given in Table S2. Pseudoviruses were purified and transduced as described [13], [40]. Transient suppression of endogenous ANGPTL4 expression in human keratinocytes was performed using either siGLO control or ON-TARGETplus SMARTpool ANGPTL4 siRNA (Dharmacon; L-007807-00) by means of DharmaFECT1.

### Transient Transfection

Human keratinocytes were transfected with cDNA encoding for MKK7-JNK or co-transfected with TAM67 as previously described. The expression levels of ANGPTL4 and transglutaminase type 1 were determined by real-time PCR. The expression vectors for MKK7-JNK and TAM67 were kind gift from R.J. Davis (University of Massachusetts Medical School, Worcester) and D.J. Templeton (University of Virginia Medical School, Charlottesville).

### Total RNA extraction and Real-time PCR

Total RNA was purified from homogenized tissues and OTCs using RNAeasy kit (Qiagen). Five µg total RNA was reverse transcribed with oligo-dT primers and qPCR was performed as previously described [21], [22]. Melt curve analysis was included to assure that only one PCR product was formed. Primers were designed to generate a PCR amplification product of 100–250 bp. Only primer pairs yielding unique amplification products without primer dimer formation were subsequently used for real-time PCR assays. Expression was related to the control gene ribosomal protein L27 (L27), which did not change under any of the experimental conditions studied. Primer sequences for real-time PCR are provided in Table S3.

### Chromatin immunoprecipitation (ChIP)

ChIP was performed as previously described [40], except that anti-p(Ser63)-c-JUN and anti-p(S79)JUNB antibodies were used. The sequence of the ChIP primers was as given in Table S1.

### Immunoblot analysis

Epidermis was physically separated from OTC after a 20-min treatment with dispase. Fibroblasts embedded in collagen were isolated after collagenase treatment. For Western blotting, protein extracts were made in ice-cold lysis buffer (20 mM Na_2_H_2_PO_4_, 250 mM NaCl, 1% Triton X-100, and 0.1% SDS). Equal amounts of protein extracts (50 µg) were resolved by SDS-PAGE and electrotransferred onto PVDF membranes. Membranes were processed as described by the manufacturer of antibodies, and proteins were detected by chemiluminescence (Millipore). Coomassie blue-stained membrane or β-tubulin was used to check for equal loading and transfer. Membrane was also stripped and reprobed with another antibody as described [40], [41].

### Statistical analysis

Statistical analyses were performed using two-tailed Mann-Whitney tests with SPSS software. All statistical tests were two-sided. p value of ≤0.05 is considered significant.

## Supporting Information

Table S1 Genes down-regulated in mouse skin of ANGPTL4^−/−^ when compared with ANGPTL4^+/+^.
(DOC)

Table S2 Oligonucleotide sequences of siRNAs and ChIP primers.
(DOC)

Table S3 Oligonucleotide sequences of real-time PCR primers.
(DOC)

## References

[1] Dotto GP (1999). Signal transduction pathways controlling the switch between keratinocyte growth and differentiation.. Crit Rev Oral Biol Med.

[2] Eckert RL, Crish JF, Robinson NA (1997). The epidermal keratinocyte as a model for the study of gene regulation and cell differentiation.. Physiol Rev.

[3] Eckert RL, Welter JF (1996). Transcription factor regulation of epidermal keratinocyte gene expression.. Mol Biol Rep.

[4] Hanley K, Devaskar UP, Hicks SJ, Jiang Y, Crumrine D (1997). Hypothyroidism delays fetal stratum corneum development in mice.. Pediatr Res.

[5] Kömüves LG, Hanley K, Jiang Y, Elias PM, Williams ML (1998). Ligands and activators of nuclear hormone receptors regulate epidermal differentiation during fetal rat skin development.. J Invest Dermatol.

[6] Michalik L, Desvergne B, Tan NS, Basu-Modak S, Escher P (2001). Impaired skin wound healing in peroxisome proliferator-activated receptor (PPAR)alpha and PPARbeta mutant mice.. J Cell Biol.

[7] Tan NS, Michalik L, Noy N, Yasmin R, Pacot C (2001). Critical roles of PPAR beta/delta in keratinocyte response to inflammation.. Genes Dev.

[8] Tan NS, Michalik L, Desvergne B, Wahli W (2003). Peroxisome proliferator-activated receptor (PPAR)-beta as a target for wound healing drugs: what is possible?. Am J Clin Dermatol.

[9] Tan NS, Michalik L, Desvergne B, Wahli W (2004). Peroxisome proliferator-activated receptor-beta as a target for wound healing drugs.. Expert Opin Ther Targets.

[10] Schmuth M, Haqq CM, Cairns WJ, Holder JC, Dorsam S (2004). Peroxisome proliferator-activated receptor (PPAR)-beta/delta stimulates differentiation and lipid accumulation in keratinocytes.. J Invest Dermatol.

[11] Tan NS, Icre G, Montagner A, Bordier-ten-Heggeler B, Wahli W (2007). The nuclear hormone receptor peroxisome proliferator-activated receptor beta/delta potentiates cell chemotactism, polarization, and migration.. Mol Cell Biol.

[12] Tan NS, Michalik L, Desvergne B, Wahli W (2005). Genetic- or transforming growth factor-beta 1-induced changes in epidermal peroxisome proliferator-activated receptor beta/delta expression dictate wound repair kinetics.. J Biol Chem.

[13] Chong HC, Tan MJ, Philippe V, Tan SH, Tan CK (2009). Regulation of epithelial-mesenchymal IL-1 signaling by PPARbeta/delta is essential for skin homeostasis and wound healing.. J Cell Biol.

[14] Kersten S, Mandard S, Tan NS, Escher P, Metzger D (2000). Characterization of the fasting-induced adipose factor FIAF, a novel peroxisome proliferator-activated receptor target gene.. J Biol Chem.

[15] Oike Y, Akao M, Kubota Y, Suda T (2005). Angiopoietin-like proteins: potential new targets for metabolic syndrome therapy.. Trends Mol Med.

[16] Mandard S, Zandbergen F, van Straten E, Wahli W, Kuipers F (2006). The fasting-induced adipose factor/angiopoietin-like protein 4 is physically associated with lipoproteins and governs plasma lipid levels and adiposity.. J Biol Chem.

[17] Sukonina V, Lookene A, Olivecrona T, Olivecrona G (2006). Angiopoietin-like protein 4 converts lipoprotein lipase to inactive monomers and modulates lipase activity in adipose tissue.. Proc Natl Acad Sci U S A.

[18] Lichtenstein L, Mattijssen F, de Wit NJ, Georgiadi A, Hooiveld GJ (2010). Angptl4 Protects against Severe Proinflammatory Effects of Saturated Fat by Inhibiting Fatty Acid Uptake into Mesenteric Lymph Node Macrophages.. Cell Metab.

[19] Clement LC, Avila-Casado C, Macé C, Soria E, Bakker WW (2011). Podocyte-secreted angiopoietin-like-4 mediates proteinuria in glucocorticoid-sensitive nephrotic syndrome.. Nat Med.

[20] Zhu P, Tan MJ, Huang RL, Tan CK, Chong HC (2011). Angiopoietin-like 4 protein elevates the prosurvival intracellular O2(-):H2O2 ratio and confers anoikis resistance to tumors.. Cancer Cell.

[21] Goh YY, Pal M, Chong HC, Zhu P, Tan MJ (2010). Angiopoietin-like 4 interacts with integrins beta1 and beta5 to modulate keratinocyte migration.. Am J Pathol.

[22] Goh YY, Pal M, Chong HC, Zhu P, Tan MJ (2010). Angiopoietin-like 4 interacts with matrix proteins to modulate wound healing.. J Biol Chem.

[23] Oliver WR, Shenk JL, Snaith MR, Russell CS, Plunket KD (2001). A selective peroxisome proliferator-activated receptor delta agonist promotes reverse cholesterol transport.. Proc Natl Acad Sci U S A.

[24] Mandard S, Zandbergen F, Tan NS, Escher P, Patsouris D (2004). The direct peroxisome proliferator-activated receptor target fasting-induced adipose factor (FIAF/PGAR/ANGPTL4) is present in blood plasma as a truncated protein that is increased by fenofibrate treatment.. J Biol Chem.

[25] Köster A, Chao Y, Mosior M, Ford A, Gonzalez-DeWhitt P (2005). Transgenic angiopoietin-like (angptl)4 overexpression and targeted disruption of angptl4 and angptl regulation of triglyceride metabolism.. Endocrinology.

[26] Maas-Szabowski N, Szabowski A, Stark HJ, Andrecht S, Kolbus A (2001). Organotypic cocultures with genetically modified mouse fibroblasts as a tool to dissect molecular mechanisms regulating keratinocyte growth and differentiation.. J Invest Dermatol.

[27] Shearer BG, Steger DJ, Way JM, Stanley TB, Lobe DC (2008). Identification and characterization of a selective peroxisome proliferator-activated receptor beta/delta (NR1C2) antagonist.. Mol Endocrinol.

[28] Rutberg SE, Saez E, Glick A, Dlugosz AA, Spiegelman BM (1996). Differentiation of mouse keratinocytes is accompanied by PKC-dependent changes in AP-1 proteins.. Oncogene.

[29] Kamioka N, Akahane T, Kohno Y, Kuroki T, Iijima M (2010). Protein kinase C delta and eta differently regulate the expression of loricrin and Jun family proteins in human keratinocytes.. Biochem Biophys Res Commun.

[30] Schönwasser DC, Marais RM, Marshall CJ, Parker PJ (1998). Activation of the mitogen-activated protein kinase/extracellular signal-regulated kinase pathway by conventional, novel, and atypical protein kinase C isotypes.. Mol Cell Biol.

[31] Schechtman D, Mochly-Rosen D (2001). Adaptor proteins in protein kinase C-mediated signal transduction.. Oncogene.

[32] Li H, Ge C, Zhao F, Yan M, Hu C (2011). HIF-1α-activated ANGPTL4 contributes to tumor metastasis via VCAM-1/integrin β1 signaling in human hepatocellular carcinoma.. Hepatology.

[33] Stapleton CM, Joo JH, Kim YS, Liao G, Panettieri RA (2010). Induction of ANGPTL4 expression in human airway smooth muscle cells by PMA through activation of PKC and MAPK pathways.. Exp Cell Res.

[34] Lei K, Nimnual A, Zong WX, Kennedy NJ, Flavell RA (2002). The Bax subfamily of Bcl2-related proteins is essential for apoptotic signal transduction by c-Jun NH(2)-terminal kinase.. Mol Cell Biol.

[35] Bornstein P, Sage EH (2002). Matricellular proteins: extracellular modulators of cell function.. Curr Opin Cell Biol.

[36] Dutton S, Trayhurn P (2008). Regulation of angiopoietin-like protein 4/fasting-induced adipose factor (Angptl4/FIAF) expression in mouse white adipose tissue and 3T3-L1 adipocytes.. Br J Nutr.

[37] Ito Y, Oike Y, Yasunaga K, Hamada K, Miyata K (2003). Inhibition of angiogenesis and vascular leakiness by angiopoietin-related protein 4.. Cancer Res.

[38] Cazes A, Galaup A, Chomel C, Bignon M, Bréchot N (2006). Extracellular matrix-bound angiopoietin-like 4 inhibits endothelial cell adhesion, migration, and sprouting and alters actin cytoskeleton.. Circ Res.

[39] Gealekman O, Burkart A, Chouinard M, Nicoloro SM, Straubhaar J (2008). Enhanced angiogenesis in obesity and in response to PPARgamma activators through adipocyte VEGF and ANGPTL4 production.. Am J Physiol Endocrinol Metab.

[40] Tan SH, Pal M, Tan MJ, Wong MHL, Tam FU (2009). Regulation of Cell Proliferation and Migration by TAK1 via Transcriptional Control of von Hippel-Lindau Tumor Suppressor.. J Biol Chem.

[41] Yeung YG, Stanley ER (2009). A solution for stripping antibodies from polyvinylidene fluoride immunoblots for multiple reprobing.. Anal Biochem.

