# Supplementary material for: Angiopoietin-Like 4 Regulates Epidermal Differentiation
Source: PLoS One. 2011 Sep 22;6(9):e25377. doi: 10.1371/journal.pone.0025377 (PMC3178651; doi:10.1371/journal.pone.0025377)
Supplement: Table S1 — Genes down-regulated in mouse skin of ANGPTL4−/− when compared with ANGPTL4+/+. (DOC) [file pone.0025377.s001.doc]

Table S1: Genes down-regulated in mouse skin of ANGPTL4^- / -^ when compared with ANGPTL4^+/+^

| Access No. | Symbol | Description | ^a^Fold change |  |
| --- | --- | --- | --- | --- |
| NM_008343 | Igfbp3 | insulin-like growth factor binding protein 3 | 0.543 |  |
| NM_007388 | Acp5 | acid phosphatase 5, tartrate resistant | 0.537 |  |
| NM_007921 | Elf3 | E74-like factor 3 | 0.536 |  |
| NM_013800 | Barx2 | BARX homeobox 2 | 0.52 |  |
| NM_027552 | Kynu | kynureninase (L-kynurenine hydrolase) | 0.518 |  |
| NM_144800 | Mtss1 | metastasis suppressor 1 | 0.512 |  |
| NM_009235 | Sox15 | SRY (sex determining region Y)-box 15 | 0.51 |  |
| NM_010801 | Mlf1 | Myeloid leukemia factor 1 | 0.51 |  |
| NM_016872 | Vamp5 | Vesicle-associated membrane protein 5 (myobrevin) | 0.51 |  |
| NM_013864 | Ndrg2 | NDRG family member 2 | 0.51 |  |
| NM_053007 | Cntf | Ciliary neurotrophic factor | 0.5 |  |
| NM_013822 | Jag1 | jagged 1 | 0.497 |  |
| NM_007928 | Mark2 | MAP/microtubule affinity-regulating kinase 2 | 0.49 |  |
| NM_145603 | Ces2 | carboxylesterase 2 | 0.482  differentiation |  |
| NM_027085 | Clic3 | chloride intracellular channel 3 | 0.462 |  |
| NM_009668 | Bin1 | Bridging integrator 1 | 0.46 |  |
| NM_019684 | Srpk3 | SFRS protein kinase 3 | 0.46 |  |
| NM_172398 | Akr1b10 | RIKEN cDNA 2310005E10 gene | 0.457 |  |
| NM_010730 | Anxa1 | Annexin A1 | 0.45 |  |
| NM_019645 | Pkp1 | plakophilin 1 | 0.442 |  |
| NM_030743 | Rnf114 | Ring finger protein 114 | 0.43 |  |
| NM_133643 | Edaradd | EDAR-associated death domain | 0.43 |  |
| NM_007631 | Ccnd1 | Cyclin D1 | 0.41 |  |
| NM_019568 | Cxcl14 | chemokine (C-X-C motif) ligand 14 | 0.392 |  |
| NM_010017 | Dag1 | dystroglycan 1 | 0.367 |  |
| NM_019662 | Rrad | Ras-related associated with diabetes | 0.318 |  |
| NM_007614 | Ctnnb1 | Catenin (cadherin-associated protein), beta | 0.24 |  |
| NM_010054 | Dlx2 | Distal-less homeobox 2 | 0.16 |  |
| NM_011468 | Sprr2a | small proline-rich protein 2A | 0.12 |  |
| NM_009871 | Cdk5r1 | cyclin-dependent kinase 5,  regulatory subunit (p35) 1 | 0.534 |  |
| NM_013614 | Odc1 | ornithine decarboxylase, structural 1 | 0.527 |  |
| NM_008009 | Fgfbp1 | fibroblast growth factor binding  protein 1 | 0.489 |  |
| NM_007633 | Ccne1 | cyclin E1 | 0.481 |  |
| NM_009876 | Cdkn1c | cyclin-dependent kinase inhibitor 1C (P57) | 0.474  proliferation |  |
| NM_008562 | Mcl1 | myeloid cell leukemia sequence 1 | 0.471 |  |
| NM_007635 | Ccng2 | cyclin G2 | 0.466 |  |
| NM_177603 | Frat2 | frequently rearranged in advanced T-cell lymphomas 2 | 0.452 |  |
| NM_008655 | Gadd45b | growth arrest and DNA-damage-  inducible 45 beta | 0.44 |  |
| NM_013471 | Anxa4 | annexin A4 | 0.417 |  |
| AK087259 | Anxa2 | Annexin A2 | 0.41 |  |
| NM_009427 | Tob1 | transducer of ErbB-2.1 | 0.324 |  |
| NM_010828 | Cited2 | Cbp/p300-interacting transactivator, with Glu/Asp-rich carboxy-terminal domain 2 | 0.525 |  |
| NM_010691 | Lbx1 | ladybird homeobox homolog 1 (Drosophila) | 0.523  transcription factors |  |
| NM_008036 | Fosb | FBJ osteosarcoma oncogene B | 0.386 |  |
| NM_013800 | Barx2 | BarH-like homeobox 2 | 0.386 |  |
| AF201289 | Tsc22d3 | TSC22 domain family 3 | 0.357 |  |
| NM_007498 | Atf3 | activating transcription factor 3 | 0.204 |  |
| NM_010638 | Klf9 | Kruppel-like factor 9 | 0.193 |  |
| ^a^ Two-tailed Mann-Whitney test was performed with *p* < 0.05. Access No. refers to the GeneBank accession. | | | | |
