# Supplementary material for: Angiopoietin-Like 4 Regulates Epidermal Differentiation
Source: PLoS One. 2011 Sep 22;6(9):e25377. doi: 10.1371/journal.pone.0025377 (PMC3178651; doi:10.1371/journal.pone.0025377)
Supplement: Table S2 — Oligonucleotide sequences of siRNAs and ChIP primers. (DOC) [file pone.0025377.s002.doc]

| Table S2: Oligonucleotide sequences of siRNAs and ChIP primers. | | |
| --- | --- | --- |
| siRNA | Sequence (5’-3’) | |
| ANGPTL4 siRNA | sense | AAAGCTGCAAGATGACCTCAGATGGAGGCTG |
|  | anti-sense | AAAAGGCTTAAGAAGGGAATCTTCTGGAAGAC |
| Control siRNA | sense | AAAGCTGTCTTCAAGATTGATATCGAAGACTA |
|  | anti-sense | AAAATAGTCTTCGATATCAAGCTTGAAGACA |
| ChIP | Sequence (5’-3’) | |
| transglutaminase type 1 | forward | TTGAGGTCCTGAGCTGAGTGTCTGC |
|  | reverse | GCTGCGGATTTGTGCATCACTG |
| involucrin | forward | CACTGCCCTCTTTCCCACACCCTAG |
|  | reverse | ATGCCCTTGTGCTCTGCTGCTGAC |
