# Supplementary material for: Angiopoietin-Like 4 Regulates Epidermal Differentiation
Source: PLoS One. 2011 Sep 22;6(9):e25377. doi: 10.1371/journal.pone.0025377 (PMC3178651; doi:10.1371/journal.pone.0025377)
Supplement: Table S3 — Oligonucleotide sequences of real-time PCR primers. (DOC) [file pone.0025377.s003.doc]

Table S3: Oligonucleotide sequences of real-time PCR primers.

| Access No. | Forward (5’ -> 3’) | Reverse (5’ -> 3’) |
| --- | --- | --- |
| NM_008343 | CCAGGAAACATCAGTGAGTCC | GGATGGAACTTGGAATCGGTCA |
| NM_007388 | CACTCCCACCCTGAGATTTGT | CATCGTCTGCACGGTTCTG |
| NM_007921 | GCTGCCACCTGTGAGATCAG | GTGCCAAAGGTAGTCGGAGG |
| NM_013800 | GCAGCTCATGGGCCTAGAG | GCGCCCCTTAGGTTTTGTG |
| NM_027552 | GTCAAGCCTGCGTTAGTGG | GGAGGGTTTGAAATTCGGAATCC |
| NM_144800 | ATGGAGGCTGTGATCGAGAAG | TCCGGCTTTGTTTATGAAGTCTT |
| NM_009235 | TGGAGGAGGCTAAGCGTCTT | CTTCTTGGCTAAAGGGGACAG |
| NM_010801 | ACCCCTTCTTCGCTGATTCTT | CTCGACGATTATGGGTTCTTCC |
| NM_016872 | GACGAAGTGACGGAAATCATGC | CTGAAGGCCGAACTCATGTCC |
| NM_013864 | TGTTGCCAGGACAAACACCC | AAAAGTGACCGAGCCATAAGG |
| NM_053007 | TCTGTAGCCGCTCTATCTGG | GGTACACCATCCACTGAGTCAA |
| NM_013822 | AAAGTGTGCCTCAAGGAGTATCA | TGAGATTGAAGGTGTTACCCCC |
| NM_007928 | TTTCGCCAGATAGTGTCTGCT | CCACAGAAAGTATCCAGCTTGTT |
| NM_145603 | TCCAGTATCGTCTGGGTGTCC | CCTCCAAAGTGAGCGATGTTC |
| NM_027085 | CATCCTGCTGTACGATGGGG | TGTGTTGGACTCCCTGTACCT |
| NM_009668 | CCTCCCAAACACACCCCATC | GAAGGGGCTCGAAGTCCAG |
| NM_019684 | GGTGGTTACTACCCAGTGAAGA | TTGCGCTGAATATCCCAGCAG |
| NM_172398 | CTAGTGCCAAACCAGAGGACC | TCCTGTATTCGAGAAGGTGTCA |
| NM_010730 | AGATCAAGGCCGCGTACTTAC | GCTGGAGTTTTTAGCATAGCCA |
| NM_019645 | ACCGTCAAACGGCAGAAGTC | AGGGTCCCATTGTAGATCGGA |
| NM_030743 | CGAGAGCATAGAGACTTCTTGC | TGGCCTTTACACCTTCCATGA |
| NM_133643 | TCACCCAACCGTGAAGAACTG | TGGCTGTTTCGGAATAAGAACTC |
| NM_007631 | CAGAAGTGCGAAGAGGAGGTC | TCATCTTAGAGGCCACGAACAT |
| NM_019568 | GAAGATGGTTATCGTCACCACC | CGTTCCAGGCATTGTACCACT |
| NM_010017 | CCAGTGTGTTCTCTATCGAGGT | CACAGGCAGATGGCACTACC |
| NM_019662 | GTCAGAGGAGGGCGTTTACAA | TCCACAGTGATAGAACGGTCA |
| NM_007614 | CCCAGTCCTTCACGCAAGAG | CATCTAGCGTCTCAGGGAACA |
| NM_010054 | GGCTCCTACCAGTACCACG | CGTTGTTGACCGGAGACGAA |
| NM_011468 | GGTGAAGGAGAACGACCAGAAG | TCAACTGTACCCAGGTGCCC- |
| NM_009871 | GACCAGGGTTTCATCACACCG | GGCTTCCTTACAGCTCTCTACC |
| NM_013614 | GACGAGTTTGACTGCCACATC | CGCAACATAGAACGCATCCTT |
| NM_008009 | CTAAATCTCTGACGCATGGCA | GAAAACTCCTGATCGGCTTGT |
| NM_007633 | GTGGCTCCGACCTTTCAGTC | CACAGTCTTGTCAATCTTGGCA |
| NM_009876 | CGAGGAGCAGGACGAGAATC | GAAGAAGTCGTTCGCATTGGC |
| NM_008562 | TCAAAGATGGCGTAACAAACTGG | CCCGTTTCGTCCTTACAAGAAC |
| NM_007635 | AGGGGTTCAGCTTTTCGGATT | AGTGTTATCATTCTCCGGGGTAG |
| NM_177603 | AGGCCGTCGGAGAAAACTTAA | GGCCCGGGAAATTCTCAT |
| NM_008655 | CAGATTCACTTCACCCTGATCC | GTTGTGCCCAATGTCTCCG |
| NM_013471 | GGAGGTACTGTCAAAGCTGC | GCCACTCAGTTCTGACTTCAG |
| AK087259 | GTGGATGAGGTCACCATTGTC | GTCGGTTCCTTTCCTCTTCAC |
| NM_009427 | CAATCTGCCACAGGATTTGAGT | TACTGTCGTCCACGTACAGCA |
| NM_010828 | GGCCGCCAGGTTTAACAACT | GGTGCAAATCCGGCATGTAG |
| NM_010691 | CGGGACCTAGAGGAGATGAAG | GAGAACCCGGCCTAGACTTAG |
| NM_008036 | ACCAGCTACTCAACCCCAG | GGGTAAGTGTCTCTTCTCGGG |
| NM_013800 | AGCAGAAGTATTTGTCTACCCCA | GCGCCCCTTAGGTTTTGTG |
| AF201289 | CGTGAGCAACTTTCGGCAG | AGAGCATGGTCTGGTCTATGT |
| NM_007498 | CAGACCCCTGGAGATGTCAGT | TTCTTGTTTCGACACTTGGCA |
| NM_010638 | CGAGCGGCTGCGACTACCTG | GGGCTGTGGGAAGGACTCGAC |

All nucleotides and probes synthesized by Sigma-Aldrich. Melting curve analysis was performed to assure that only one PCR product was formed. Primers were designed to generate a PCR amplification product of 100 to 250 bp. Only primer pairs yielding unique amplification products without primer dimer formation were subsequently used for real-time PCR assays.
